# Elevated production of radical oxygen species by polymorphonuclear neutrophils in cerebrospinal fluid infection

**DOI:** 10.1186/2110-5820-2-10

**Published:** 2012-04-10

**Authors:** Anne-Claire Lukaszewicz, Géraldine Gontier, Valérie Faivre, Ingrid Ouanounou, Didier Payen

**Affiliations:** 1 Department of Anesthesiology and Critical Care, Lariboisière Hospital, Univ Paris Diderot, 2 rue Ambroise Paré, Paris cedex 10, Sorbonne Paris Cité 75475, France

**Keywords:** Meningitis, Diagnosis, Nosocomial, External ventricular drain, Neurointensive care, Reactive oxygen species

## Abstract

**Background:**

Central nervous system infection is a daily concern in neurointensive care; however, diagnosis remains difficult because classical criteria based on cerebrospinal fluid (CSF) analysis are difficult to interpret in post-trauma or neurosurgery patients after recent bleeding. A rapid, specific, sensitive test to diagnose CSF infection would help streamline therapeutic decisions in clinical practice and limit the risk of multiresistant bacteria. We hypothesized that polymorphonuclear neutrophil (PMN) phenotype and radical oxygen species (ROS) production in CSF may be specific to the presence of infection.

**Methods:**

This study included 30 patients with suspected CSF infection with ventricular hemorrhage requiring external ventricular drainage, and 13 patients after trauma or surgery. Criteria for evaluating CSF infection included positive culture and > 100 leukocytes/mm^3^. Analysis of PMN phenotype was performed using flow cytometry (CD16, CD11b, and CD62L). ROS production was analyzed through luminometry (luminol).

**Results:**

Infected CSF exhibited higher production of ROS compared with noninfected CSF. PMNs in CSF exhibited low CD16 and high annexin V expression, suggesting apoptosis.

**Conclusions:**

Measurement of ROS production may discriminate infected from noninfected CSF. This simple test would be easy to employ in clinical practice to improve CSF infection management.

## Background

Diagnosis of meningeal or intracranial infection remains difficult in post-trauma or neurosurgical patients for several reasons. Clinical symptoms often are mild and nonspecific and sometimes are masked by corticoids or therapeutic hypothermia. Additionally, classical criteria based on cerebrospinal fluid (CSF) analysis, such as pleocytosis with a high proportion of polymorphonuclear neutrophils (PMNs), low glucose, and high protein levels, are difficult to interpret soon after bleeding or surgical procedures. Finally, direct bacteriological examination results may be negative because of concomitant antibiotic therapy for another treated infection [1]. Because such infections can have a major impact on patient evolution and be detrimental to prognosis, clinicians have no choice but to perform repeated CSF analyses and to administer broad-spectrum antibiotics for at least 48 to 72 h while awaiting validation by microbial cultures. Such risk-based care leads to repeated CSF checking and promotes the selection of antibiotic-resistant organisms [2]. A rapid, specific, sensitive test with which to diagnose CSF infection could streamline therapeutic decisions in clinical practice.

Ventriculostomy catheterization, or external ventricular drainage (EVD), is an important and frequently used invasive procedure in neurosurgical and intensive care practice. CSF drainage is a key component in the treatment of acute brain injury and represents the mainstay of emergency treatment for hydrocephalus, especially in cases of ventricular hemorrhage. However, it is an invasive procedure that can expose the patient to CNS infection [3]; in fact, the most common complication of EVD is infection (ventriculo-meningitis), with an incidence of close to 10% [3]. High risk of infection has been associated with intraventricular hemorrhage and the duration that the catheter remains in situ [4].

PMNs are the first line of defense in the innate immune response to bacterial infection, and their activated phenotype may constitute a hallmark of infection [5]. The killing system of the activated PMNs includes the production of radical oxygen species (ROS), also called respiratory burst [6]. NADPH oxidase plays a pivotal role in this process by pumping superoxide ions (O_2_^-^) into phagocytic vacuoles. Superoxide production and its reaction products are collectively referred to as ROS; they can be detected and measured by luminescence or by fluorescence after the oxidation of reactive components [7-9].

We hypothesized that PMN phenotype and level of ROS production may be altered in infected CSF compared with noninfected CSF. In the present study, we studied PMN activation markers and ROS production in CSF from patients with EVD according to status of infection. We also applied this test in CSF obtained by lumbar puncture after trauma or surgery. Finally, we studied the stability of ROS measurements under different experimental conditions relevant to clinical practice.

## Methods

This study was approved by the ethical committee of our institution (IRB 00006477). The study included patients who were admitted to the intensive care unit (17 beds) of our teaching hospital during a 1-year period, particularly patients with ventricular hemorrhage requiring EVD. The physician in charge was not aware of the study investigations, and patient management was independent of the results. CSF samples were treated rapidly (within 2 h) in the laboratory of the Department of Critical Care and Anesthesia and analyzed by flow cytometry in the laboratory of hematology, both in the hospital.

### Definitions of CSF infection

Four different categories were used to describe CSF infection status: (1) CSF was considered "infected" (ventriculo-meningitis) when it was positive for bacterial infection upon direct examination or in culture and contained > 100 leukocytes/mm^3^; (2) CSF was considered "noninfected" if it contained > 100 leukocytes/mm^3 ^but the results of direct examination and culture were negative after 72 h; (3) CSF was considered "negative" if the leukocyte count was < 100 leukocytes/mm^3 ^and the results of direct examination and cultures were negative after 72 h [2,10]; (4) CSF was considered contaminated when it was positive for bacterial infection upon direct examination or in culture but contained < 100 leukocytes/mm^3^.

### Clinical and biological data collection

For each patient, we collected data regarding demographic characteristics, indication for EVD, classical clinical signs of inflammation and CSF analyses (cell count, protein, and glucose levels), treatment regimen at the time of sampling, and outcome. We also noted other concomitant sites of infection and antibiotic regimen, if any.

### Measurements of ROS production by luminometry

CSF (250 μl) was diluted in Hanks' Balanced Salt Solution (HBSS; Invitrogen, Cergy-Pontoise, France) to a final volume of 1 ml and incubated with luminol (50 μM; Sigma) for 10 min at 37°C in the dark. As a functional test, a control was compared with a sample that had been stimulated with 10^-7 ^M of phorbol 12-myristate 13-acetate (PMA; Sigma); each condition was assayed in duplicate. Immediate analysis was conducted during a 20-min period using a luminometer (AutoLumat *Plus *LB 953; Berthold Technologies, Bad Wildbad, Germany). The signal was recorded for a duration of 1 second every minute and was recorded as relative light units (RLU). Results were expressed as area under the curve (AUC) of luminescence during the 20 min.

For some samples, we performed several measurements to test reproducibility of the luminometric analysis under different conditions that might be encountered in clinical practice. We chose three different experimental conditions: (1) measurements after a 2-h wait at room temperature; (2) after a 2-h wait at 4°C; and (3) after centrifugation (10 min, 375 × *g*, room temperature) and cell washing in HBSS. Measurements obtained in blood PMNs from healthy volunteers with the same protocol (40 μl) were considered reference values for basal PMN parameters.

### Characterization of PMNs by flow cytometry

After centrifugation (200 × *g*, 10 min, room temperature), CSF cells were resuspended in 50 μl of CSF and incubated in the presence of specific antibodies for 30 min in the dark in a 37°C water bath. Anti-CD16-Phycoerythrine-Cyanine 5 (PC5) antibody (Beckman Coulter, Marseille, France) was used for PMN selection, and phenotype was assessed using anti-CD11b-Phycoérythrine (PE) or anti-CD62L-PE (BD Bioscience, San Jose, CA). Nonspecific binding of antibodies to cell Fc receptors was assessed with a control isotype. Red cells were eliminated after incubation with lysing solution (BD FACS Lysing Solution; BD Bioscience) for 10 min in the dark at room temperature and washing with phosphate buffered saline (PBS; Gibco Invitrogen, Grand Island, NY). Next, the technical procedure was performed according to the manufacturer's recommendations until the flow cytometric analysis (FacsCanto, and FacsDiva software; Becton Dickinson). The Quantitative Flow CytoMetry flowmeter was used to convert mean fluorescence intensity into AB/C (Quantibrite^®^, Becton, Dickinson and Company). The regression slope of correlation between mean fluorescence intensity and AB/C never varied over time and ranged between 0.999 and 1.

Some samples were used to quantitate apoptosis by annexin-V measurement (apoptosis detection kit; Sigma, Saint Quentin Fallavier, France). This technical procedure was performed according to the manufacturer's recommendations.

### Statistical analysis

Quantitative variables were expressed as median and interquartile range (IQR). Analysis was performed using nonparametric Mann-Whitney and Kruskal-Wallis tests; when significant (*P *< 0.05), 2-by-2 between-group comparison was performed.

## Results

### Patients with suspected CSF infection

During a 1-year period, 30 patients required EVD because of intraventricular hemorrhage with a variety of causes: subarachnoid hemorrhages by aneurysm rupture (n = 20), parenchymal hematoma (n = 6), operated tumors (n = 3), and severe brain trauma (n = 1). Patients' mean age was 46 (range, 44-52) years, and they presented with a mean SAPSII of 42 (range, 34-49) at admission. Four patients died during the study of causes related to the severity of their intracranial vascular pathology. During the same period of time, we also tested CSF samples obtained by lumbar puncture from patients with suspected infection a mean of 8 (range, 7-12) days after trauma or surgery (n = 2 subarachnoid hemorrhages by aneurysm rupture, n = 1 parenchyma hematoma, n = 5 operated tumors, and n = 6 severe brain trauma).

### Global ROS production in CSF from patients with suspected infection

According to the protocol, CSF was sampled by EVD in response to fever (> 38°C). Fifteen patients met the criteria for noninfected CSF, seven for infected CSF, and eight for negative CSF. The microorganisms observed in the seven infected CSF cases were *Pseudomonas aeruginosa, Staphylococcus aureus, Staphylococcus epidermidis, Enterococcus faecalis, Acinetobacter baumanii*, and *Corynebacterium*. Table 1 summarizes the patients' clinical and biological characteristics. Ventricular drainage continued for a longer duration in patients with infected CSF. According to the definition, cell count in CSF was higher in "infected" and "noninfected" groups than in the "negative" group; the highest count was recorded in the infected group. No difference in percentage of PMNs was reported between groups.

**Table 1:** Clinical and biological variables, separated by CSF inflammation and infection status

	Negative n = 8	Noninfected n = 15	Infected n = 7	*P *value	*P1*	*P2*	*P3*
Temperature°C	39.0(38.5-39.3)	39.4(39.0-39.8)	39.5(38-40.3)	0.4557			

Blood leukocytes10^9^/mm^3^	13.4(10.3-18)	12.5(10.1-16.2)	19.1(12.5-21.1)	0.9158		0.0039	

Blood glucosemmol/L	6.5(5.4-7.3)	6.9(6.4-7.6)	8(7.1-8.5)	0.2496			

Delaydays	5(4-7)	7(5-10)	11(10-18)	0.0027	0.049	0.0026	0.0165

CSF leukocytes/mm^3^	32(6-73)	360(170-1022)	460(190-1090)	0.0002	0.0001	0.0012	

CSF % PMN	83(65-89)	83(74-93)	88(83-93)	0.6663			

CSF glucosemmol/L	3.6(3.0-3.9)	3.7(3.3-4.4)	2(0.9-3.9)	0.3013			

CSF proteinsg/L	0.7(0.5-0.7)	0.6(0.4-0.8)	1.5(1.1-2.3)	0.0042		0.0039	0.0045

*P *value comparison among the three groups (Kruskall-Wallis). Between-group comparison: *P1 *value negative versus noninfected, *P2 *value negative versus infected, *P3 *value noninfected versus infected groups (Mann-Whitney test). Data are expressed as median (interquartile range)

**Figure 1:**
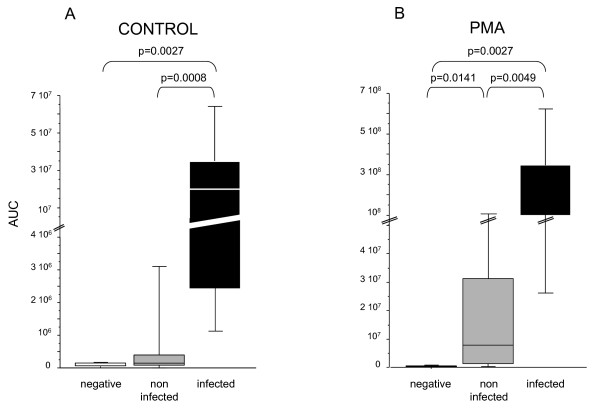
**Quantity of ROS produced by cells in 250 μl of cerebrospinal fluid (CSF) under basal conditions (A) or after stimulation by phorbol 12-myristate 13-acetate (PMA) (B)**.

**Figure 2:**
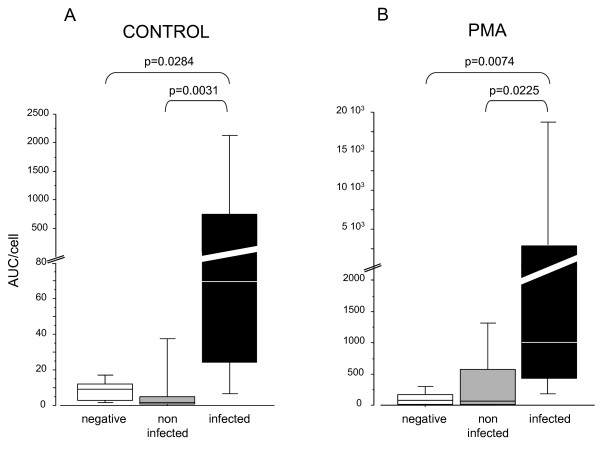
**Quantity of ROS normalized per cell under basal conditions (A), and after PMA stimulation (B)**. Samples were grouped according to infection status: negative CSF (white, n = 8), noninfected CSF (gray, n = 15), and infected CSF (black, n = 7).

**Figure 3:**
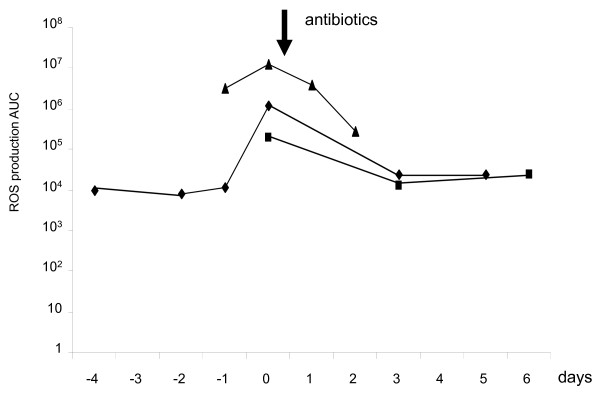
**Repetitive measurement of ROS in 250 μl of cerebrospinal fluid on several days: follow-up in three patients and impact of antibiotic treatment**. The arrow represents the start of antibiotic treatment (day 0).

At the basal state, ROS production was significantly higher in the infected group than in the noninfected and negative groups (Figure 1A). After stimulation with PMA (Figure 1B), higher ROS production was observed in CSF from the infected group compared with the negative and noninfected groups. Basal ROS production did not differ between the noninfected group and the negative group; however, it was higher in the noninfected group than in the negative group after PMA stimulation. When ROS production values were normalized to PMN number (per cell; Figure 2), we observed the same trends, but with smaller differences. To validate the sensibility of using ROS to diagnose infection, we reported the follow-up in three patients after treatment with antibiotics and demonstrated a rapid decrease of ROS production (Figure 3).

In CSF obtained by lumbar puncture, the leukocyte count was higher in infected samples (2590/mm^3^, IQR 1295-2925) than in noninfected (1200/mm^3^, IQR 943-4828; *P *= 0.0233) and in negative samples (20/mm^3^, IQR 13-35; *P *= 0.0339). Noninfected CSF had a lower leukocyte count than negative samples (*P *= 0.0167). ROS measured in CSF differed significantly among the three groups: negative CSF (75.5 × 10^3^, IQR 49.0 × 10^3^-203.4 × 10^3^); noninfected CSF (5,760 × 10^3^, IQR 3,545 × 10^3^-11,582.5 × 10^3^); and infected CSF (52,500 × 10^3^, IQR 23,700 × 10^3^-84,050 × 10^3^; *P *= 0.0073, Kruskal-Wallis test). A similar pattern was observed when the leukocyte counts were normalized to PMN number and after PMA stimulation (data not shown).

### PMN phenotype in noninfected and infected CSF during EVD

**Figure 4:**
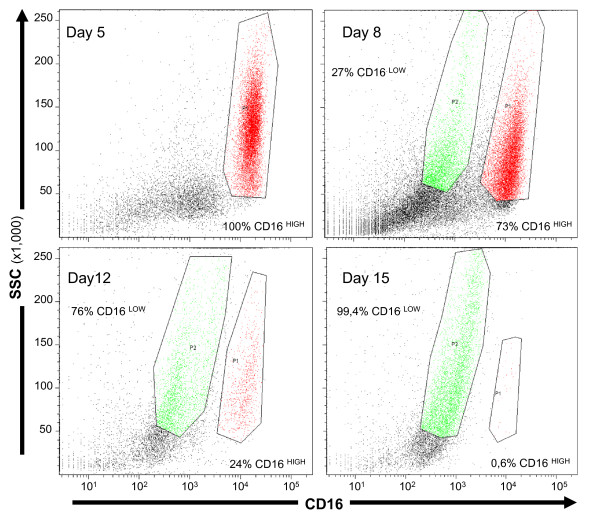
**CD16 expression on polymorphonuclear neutrophils in cerebrospinal fluid over time in the same patient**. Two cell populations are distinguished by differing CD16 expression and granulosity (SSC): CD16^high ^and CD16^low^.

**Figure 5:**
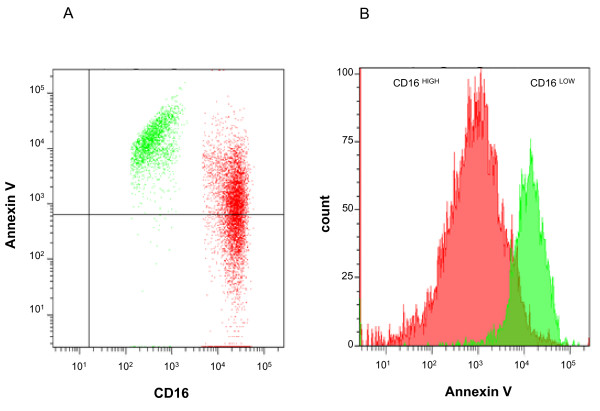
**Representative annexin V staining in the CD16^high ^and CD16^low ^polymorphonuclear neutrophil (PMN) populations**. CD16^low ^PMN exhibited the highest annexin V level.

Eleven patients with EVD were sampled for CSF PMN characterization. Flow cytometric analysis clearly revealed two distinct populations of PMNs with respect to CD16 expression: CD16^high ^and CD16^low ^cells (Figure 4). Repeated analysis of CSF over time demonstrated that the proportion of CD16^high ^cells decreased in CSF between day 5 and day 15 (Figure 4). CSF PMNs also showed increased annexin V expression at the cell surface, especially in the CD16^low ^population (Figure 5), suggesting that they became apoptotic [11].

CD11b and CD62L are adhesion molecules, and their level of expression correlates with activation status. CD11b expression was similar in PMNs from CSF (33,900 AB/C, IQR 26,200-39,100) and from blood in healthy volunteers (47,900 AB/C, IQR 17,474-77,242); the percentages of CD62L-positive PMNs were similar in CSF (32% (6-41)) and healthy blood (12% (7-22)). PMNs in CSF did not present enhanced markers of activation.

One patient presented with an infection on day 10. The infected CSF was characterized by an increased number of CD16^high ^cells, high CD11b expression (119,100 AB/C), and a low percentage of CD62L-expressing PMNs (4%). The heightened expression of CD16 and CD11b was probably related to the recruitment of new leukocytes. The presentation of PMNs with high CD11b levels and low CD62L in cases with infection was in agreement with the classical activated phenotype described in the literature [12].

### CSF analysis by luminometry under different experimental conditions

Different experimental procedures were conducted to study the impact of various experimental conditions on the results. We performed analyses at different temperatures and with different time delays before measurements. CSF samples collected during patients' follow-up were analyzed following HBSS lavage plus centrifugation, and the results were compared with those obtained without centrifugation. ROS production measured by luminometry was always higher after washing and centrifugation than without (basal AUC RLU: 16,101 (IQR 9,835-35,358) versus 76,336 (IQR 41,222-277,007), *P *< 0.0001; PMA-stimulated AUC RLU: 315,592 (IQR 90,668-1,504,078) versus 683,190 (IQR 169,492-3,676,343), *P *= 0.0002). Neither the duration of delay before sample treatment (2 versus 4 h) nor the storage temperature (4°C versus room temperature) affected ROS production under basal or PMA-stimulated conditions, as measured by luminometry.

## Discussion

Among phenotypic characteristics, ROS production may represent a hallmark of infected CSF. High spontaneous ROS production was related to increased PMN count following cell recruitment to infected tissue. The global increase in ROS production also was related to higher synthesis, because ROS production expressed "per cell" was elevated in cases of infection. ROS measurement in CSF may constitute a rapid test with which to diagnose infection in CSF and may be a suitable tool for use at bedside in clinical practice.

ROS production may be related to mitochondria production, especially in the context of apoptotic evolution [11,13-15]; however, at least some of the cells that we studied were still responsive to PMA stimulation, as viable cells would be. The main source of ROS is likely the activation of the NADPH oxidase system, as supported by the response to PMA stimulation [14,16]. This hypothesis is in agreement with the septic context, and the recruitment of reactive phagocytes might imply enzyme upregulation at the transcriptional level [17].

With the objective of developing a test that will diagnose CSF infection, the measurement of spontaneous ROS production by CSF cells seemed to be more discriminating than PMA-stimulated production, because little overlap was observed between the ranges exhibited by noninfected and infected samples. After a threshold is validated in a large-scale study and translated into clinical practice, such a test could drive the administration of antibiotics with few false-positive cases and also could help to schedule catheter changes in cases of infection. The impact of antibiotics on ROS production constitutes a criterion for the validation of the test, and its value during treatment follow-up has been demonstrated in three patients (Figure 3). However, the combination of low spontaneous and high PMA-stimulated ROS production signals the presence of numerous PMNs in the sample but without infection. In this situation, antibiotics would not be administered.

We also obtained preliminary results in CSF obtained by lumbar puncture regarding suspected postoperative or post-trauma meningitis. ROS production had the same profile between groups as was observed with CSF drainage samples, although it reached higher levels. If confirmed in a large-scale study, such overproduction of ROS might be related to the intensity of inflammation induced by surgical products and hemostatic material. We may have to consider clinical conditions when interpreting the results.

As observed by other authors, bacterial and aseptic meningitis could not be distinguished by clinical characteristics alone. This distinction was not possible with any of the classical CSF characteristics studied because of large degrees of overlap between the ranges. Lactate level in CSF may be of interest in the differential diagnosis of bacterial and viral meningitis [18] but has been inconsistently deemed relevant in the nosocomial context [5]. The specificity of inflammatory parameters is limited in postinjury or surgical contexts. The efficacy of molecular methods to determine the bacterial origin of meningitis remains to be evaluated if a rapid test is developed [2].

We measured ROS production in CSF using two different methods: (1) the reaction of intracellular hydrogen peroxide (H_2_O_2_) with DHR 123 by fluorescence using flow cytometry (data not shown), and (2) the reaction of global ROS production (primarily superoxide anion) with luminol was measured using luminescence [7]. We obtained concordant results with both methods (data not shown). Our method of choice was luminescence, which may be sensitive, specific, and simpler than DHR fluorescence measured by flow cytometry, and therefore appears to be more suitable for rapid bedside diagnosis in clinical practice.

## Conclusions

We observed that, in cases of infection, as the first line of innate immunity, newly recruited PMNs may present changes in phenotype that are specifically related to infection. As a consequence, the measurement of ROS production in CSF may be sufficient to discriminate infected from noninfected CSF.

We propose a rapid test for the diagnosis of CSF infection in the difficult context of postoperative or nosocomial meningitis. This test is based on the intensity of ROS production and appears to be discriminative enough to be used in clinical practice at bedside. Such a test could limit the use of broad-spectrum antibiotics and also might occasionally limit the use of invasive procedures, such as EVD. These are very preliminary results, and a large-scale study is needed to observe whether elevated ROS production is a hallmark of ongoing CSF infection and can be extended to other cases of postoperative and postinjury meningitis. The test could be adapted for community-acquired meningitis but only in cases with sufficient recruitment of PMNs in the CNS.

## Abbreviations

CNS: Central nervous system; CSF: Cerebrospinal fluid; EVD: External ventricular drainage; PMNs: Polymorphonuclear neutrophils; ROS: Radical oxygen species.

## Competing interests

This work was supported by the Quadrienal plan for research from Paris Diderot University and the French Ministry of Research (EA 3509). Anne-Claire Lukaszewicz and Didier Payen are holders of the patents EUROPE - n°10290421.6 (2010) and PCT/IB2011/053329 (2011).

## Authors' contributions

ACL and GG have equally contributed to the manuscript and are both considered as first author. ACL had the original idea, conceived of the experiments, designed the study, guided the experimental work, analyzed and interpreted the data, and wrote the manuscript. IO and GG performed all of the experimental work. VF participated in study design and conducted the cytometry. DP participated in study design and made major contributions to the intellectual content and writing. All authors read and approved the final manuscript.

## Authors' information

All authors work in the Department of Anesthesiology and Critical Care, Lariboisière Hospital, University of Paris Diderot, Sorbonne Paris Cité. GG was a student working toward a Master of Science degree. All authors read and approved the final mansucript.
